# The Cold War of Pictures: Framing Returning Prisoners of War in Austria’s Illustrated Press

**DOI:** 10.1080/03087298.2018.1556471

**Published:** 2019-03-14

**Authors:** Marion Krammer, Margarethe Szeless

**Keywords:** prisoners of war, postwar Austria, Cold War, propaganda, Ernst Haas (1921–86), *Heute* magazine, picture editors, Yoichi Okamoto (1915–85), Warren Trabant

## Abstract

This article examines the photographic subject of the return of Austrian prisoners of war in the aftermath of the Second World War. Scholars and curators have singled out what are known as the ‘homecomer’ photographs by the Austrian photographer Ernst Haas, praising their artistic quality and assigning these works an iconic status. This approach has obscured the broader historical background of this subject as well as the editorial practices, collaborative efforts, and propagandistic intentions of these works in the field of photojournalism. In contrast, this article focuses on the immediate historical and political context of returning prisoner-of-war photographs in the Austrian illustrated press, arguing that they were part of a broad visual discourse deliberately adopted by the postwar media for the purposes of Cold War propaganda.

This article explores the photographic depiction of returning Austrian prisoners of war (POWs) after the Second World War against the backdrop of Cold War politics in Austria – a nation which was under Allied occupation from 1945 to 1955. The four occupying forces – the Americans, the Soviets, the British, and the French – implemented media policies designed to fulfil their respective cultural and political missions. The realm of press photography and photojournalism was thus an important arena for visual propaganda as the Cold War gained traction in the fraught context of postwar Austria.

The landscape of the illustrated press in occupied Austria was composed of a wide range of different print titles whose editors represented an Austrian perspective usually affiliated with a particular political party.^1^1 – See Gertraud Lankes and Beatrix Zauninger, ‘Die frühe Illustriertenkultur der Zweiten Republik’, in Michael Schmolke, ‘Medien und Kommunikationskultur der Zweiten Republik, 3. Teilprojekt: Zeitschriften’, unpublished research paper, University of Salzburg 1989, 133–52; and Marion Krammer, ‘Rasender Stillstand oder Stunde Null? Eine Kollektivbiografie österreichischer PressefotografInnen 1945–1955’, PhD thesis, University of Vienna 2017, 25–58. There were also illustrated publications distributed by the Soviet and US occupying forces that differed in their political messages and visual quality. The following case study focuses on a theme that was to be found in abundance in all publications, irrespective of their political colour, and not least because in the context of an intensifying Cold War the subject had an emotional power that could be exploited for propaganda purposes. This article examines the visual coverage of Austrians returning from Russian prison camps and explores how the topic was co-opted by different actors in postwar occupied Austria.

It is surprising that the subject of the ‘homecomer’ – a motif absolutely central to Austrian memorial culture of the postwar era – has been the subject of so little consideration to date. The specialist literature has largely focused on the homecomer photographs of Ernst Haas to the exclusion of all others, hindering a more in-depth engagement with this subject and the wider historical context for Haas’s series. On the one hand, therefore, this article critically engages with the iconic status accorded Haas’s photographs within the literature, whilst also positioning these images within their wider historical, sociopolitical, and visual context. On the other hand, this article approaches Haas’s series as a case study for the analysis of a hitherto unexplored area of Austrian photography history.

## Ernst Haas’s Iconic Photographs

The images of prisoners returning from the Second World War are an integral part of postwar iconography in Austria. Some of these images have become iconic because they symbolise the terrors of war and, at the same time, the hopes for a new beginning. Many contemporary Austrian press photographers took pictures of returning POWs since these photographs were in high demand by the local illustrated press – a fact that has hitherto been completely ignored in photographic research. Instead, academic researchers and museum curators singled out the POW series by Haas, praising its artistic quality and proclaiming its iconic status. This approach systematically ignored the broader historical background and obscured the editorial practices, collaborative efforts, and propagandistic intentions in the field of photojournalism. The discourse about Haas’s iconicity serves here as a point of departure for raising questions about the photographic culture around the return of POWs that go beyond art historical concerns. What was the immediate historical, political, and visual context of Haas’s series?^2^2We will focus on the Austrian context and not deal with the topic of POWs returning to Germany and their photographic representation. However, a comparative study would be desirable in future. How were photographs of returning POWs used in Cold War propaganda in occupied postwar Austria?

Haas’s POW photographs were originally published on 3 August 1949 in *Heute*, an illustrated magazine edited by the US occupying forces in Munich.^3^3Ernst Haas and Inge Morath, ‘Und die Frauen warten … Die Geschichte jedes Krieges wird mit Tränen geschrieben’, *Heute*, 90 (3 August 1949), 16–23. The title of the report, ‘Und die Frauen warten … Die Geschichte jedes Krieges wird mit Tränen geschrieben’ (And the women are waiting … The story of every war is written in tears), consciously highlights the two main aspects for which Haas’s series would become famous. On the one hand, there is a focus on the fate and the anxiety of the waiting women, while, on the other, the photographs open up possibilities for identification with the general human suffering caused by every war. The narrative structure of the series, which extends over four double-page spreads of the magazine, is simple and powerful. The first photographs in the series show crowds of people, mostly women, held back by policemen.^4^4Regrettably, Ernst Haas’s reportages of returning POWs published in *Heute* magazine and *Life* magazine cannot be shown in this article since the Haas estate and Gettyimages did not grant ‘fair use’ for reproducing these images. Please visit our website ‘War of Pictures’ for viewing the double-page spreads and the layout of Haas’s famous POW reportages published in 1949 in the magazines *Heute* and *Life* at https://warofpictures.univie.ac.at/heimkehrer/medienspecial (accessed 12 December 2018). The women look anxiously and expectantly off to the right at something beyond the frame of the picture. On the next double-page spread we see an outbreak of joy: one woman must have recognised a family member; another woman happily embraces a young man. The following double page is dedicated to those still missing and culminates in a full-page photograph on the right. It shows an old lady holding up the photograph of a missing young soldier while a smiling young returnee passes by. Finally, the last double-page spread emphatically expresses the pain and sadness of those still waiting for their missing family members. The emotional impact of these photographs on those at the time must have been strong since Haas’s photographs triggered a passionate discussion about the moral boundaries of photojournalism as examined in several issues of *Photo Magazin* in 1950.^5^5Ludger Derenthal, *Bilder der Trümmer- und Aufbaujahre. Fotografie im sich teilenden Deutschland*, Marburg: Jonas 1999, 193. The POW series also earned Haas an invitation to join the renowned Magnum agency, which marks the starting point of his successful international career.^6^6Ernst Haas and Inge Morath arrived in Paris on 14 July 1949 to work for Magnum.

Like many other images from the realm of press photography, Haas’s series of returning POWs has become canonised in art history. Over recent decades, the series has regularly been shown in art museums all over the world.^7^7See the following exhibitions: ‘Ernst Haas. Heimkehrer’, Gallery Fotohof, Salzburg 1982; ‘Ernst Haas: A Photographic Retrospective on the Occasion of the Artist’s 90th Birthday’, Museum der Moderne Rupertinum, Salzburg 2011; ‘Ernst Haas – A World in Ruins: Vienna 1945–1948’, Museum der Moderne Salzburg 2005; ‘Ernst Haas in Black and White’, International Center of Photography, New York 1992; and ‘Kunst/Geschichten’, Museum der Moderne Salzburg 2014 and Museum moderner Kunst Stiftung Ludwig Wien 2018. It is examined in numerous monographs dealing with Haas’s vision and aesthetic approach and can be found in anthologies on the photographic icons of the twentieth century without any historical contextualisation.^8^8‘Ernst Haas. Wien’, in *Photo-Icons. Die Geschichte hinter den Bildern 1928–1991, vol. 2*, ed. Hans Michael Koetzle, Cologne: Taschen 2002, 64–71; Ludger Derenthal ‘“… und die Frauen warten”. Ernst Haas Kriegsheimkehrer’, in *Fotografische Leidenschaften*, ed. Katharina Sykora, Ludger Derenthal, and Esther Ruelfs, Marburg: Jonas 2006, 189–93; and Matthias Zipfel, ‘Der entscheidende Augenblick … im Pressefoto’, *Color Foto*, 11 (November 1985), 106–07. The picture of the old lady holding up the photograph of a young soldier, in particular, is part of Austria’s memorial culture of the Second World War and frequently serves as an illustration in history textbooks for Austrian schools.^9^9See ‘War of Pictures: Bildkultur in Österreich 1945–1955’, https://warofpictures.univie.ac.at, chapter 4 ‘homecomers’ (accessed 25 October 2018). This photograph has provoked many scholarly comments and interpretations.^10^10Peter Zawrel, ‘Das Erinnern vergessen. Über die Ränder der österreichischen Photographie’, in *Fisch & Fleisch. Photographie in Österreich 1945–1955*, ed. Carl Aigner, Leo Kandl, and Helmut Schäffer, Krems: Kunsthalle 1995, 12–24; Albert Lichtblau, ‘Befreit, besetzt und in Trümmern. Nach dem Ende des Zweiten Weltkrieges’, in *Ernst Haas: A World in Ruins, Vienna 1945–1948*, ed. Agnes Husslein-Arco, Weitra: Bibliothek der Provinz 2005, 37–51; and Derenthal, ‘“… und die Frauen warten”’. Analysing the viewing direction of the protagonists, the photohistorian Ludger Derenthal has written about the ‘staging of missing glances’.^11^11Derenthal, ‘“…und die Frauen warten”’, 193. The old order, symbolised by the portrait of the soldier wearing the uniform of the Wehrmacht, is deliberately ignored by the new order, symbolised by the returning POW who marches energetically into a better future and away from his involvement with National Socialism. This photograph has therefore often been interpreted as a symbol of Austria’s repression of its National Socialist past and failure to assume responsibility for it.

The origin of Haas’s POW series is comparatively well known from the photographer’s own account and from other contemporary witnesses.^12^12Ernst Haas, ‘Seit 60 Jahren lerne ich sehen’, *Geo spezial*, 2 (1982), 9; and ‘Heimkehrer. Ein Gespräch mit Ernst Haas von Michael Mauracher (part 1 + 2)’, in *Fotoseite. Kommentierte Beiträge zur Fotografie aus der Wiener Zeitung Extra*, ed. Kurt Kaindl, Salzburg: Müller 1990, 24–25. Haas’s Viennese girlfriend at the time, the actress Ernie Mangold, testified to Haas’s frequent trips to the Südbahnhof station and to his development of the POW series in their bathtub. Ernie Mangold, telephone interview, Vienna, 30 May 2011. According to Haas, he was on the lookout for a location for a fashion shoot when he stumbled upon the crowds of women waiting at the Südbahnhof train station in Vienna and immediately sensed the possibility of a great story.^13^13‘Heimkehrer (part 1)’, 24. While this personal account, given later, stresses coincidence and artistic intuition as a means to success, a closer look at the facts reveals a more complex picture.

The first transport from the Soviet Union carrying returning POWs arrived in Austria on 12 September 1947. Like many other Austrian press photographers, Haas started taking photographs of these *Heimkehrer*, or homecomers, as early as October 1947. Some of these pictures were published a month later in the Austrian magazine *Film*, edited by the Austrian actor and film producer Willi Forst.^14^14Willi Forst, ‘Der nächste Krieg…?’, *Film*, 18 (November–December 1947), 7–9. This article is illustrated with three photographs by Haas dated October 1947. One photograph shows war-damaged buildings in Vienna’s ninth district; another shows a group of people waiting expectantly at the train station in Wiener Neustadt; and the third photograph shows a reunited couple walking away into ‘a new life’, as suggested by the caption. This photograph was incorporated in Ernst Haas, ‘Last War Prisoners Come Home to Vienna’, *Life*, 90 (8 August 1949), 30–31. In January 1949, *Film* published another three of Haas’s pictures of returning POWs with the headline plea: ‘Let all come home!’^15^15‘Lasst alle heim!’, *Film*, 32 (January 1949), 6. Although the three photographs of POWs reunited with their relatives taken by Haas are not dated, the caption claims that they were taken on the occasion of a recent arrival of a POW transport, indicating that these photographs can be dated to December 1948 or January 1949. Spring 1949 also marked the beginning of Haas’s cooperation with Inge Morath, who at this time was the Austria editor for *Heute* magazine responsible for writing on Austrian topics and collecting exceptional pictorial material.^16^16Inge Morath (at the time Mörath) was employed at the Feature Section of the American Information Services Branch from August 1945 until 31 December 1947. From January 1948 until summer 1949, she worked for the Information Services Branch as the Austrian picture editor for the magazine *Heute*. See Margarethe Szeless, ‘“Das Auge lernt und wird sensibler”. Die Anfänge von Inge Moraths Karriere in Wien 1946–1949’, *Fotogeschichte*, 135 (2015), 44–47. Morath brought Haas’s POW series to the attention of *Heute*’s US editor in chief, Warren Trabant, who later gave the following testimony about Haas’s photographs:

When I saw the first set of pictures, I knew I had stumbled upon a genius and I felt a chill up and down my spine – a rare treat for someone who had shuffled through thousands of photographs, appreciating some, being impressed by others, but never really thrilled until that day.^17^17Amla Dhirajlal Sanghivi, ‘Ernst Haas’, PhD thesis, Graduate School of Syracuse University 1981, cited in Franz Simak, ‘Der Photograph Ernst Haas 1921–1986’, PhD thesis, University of Vienna 1990, 7.

Trabant’s enthusiastic account affirmed the exceptional and artistic status of Haas’s images while playing down his own role as editor. However, it is the picture editor who decides which stories are to be published and who puts forward a selection of photographs, discarding the abundance of other shots taken by the photographer. In Haas’s case, at least thirty other POW photographs are known apart from those published in *Heute*.^18^18The Museum der Moderne Salzburg holds 124 prints by Haas showing postwar Vienna 1946–48, including some of his famous POW pictures (DFL 1134/1–57, F 1135/1–57, and DFL 221/1–10). In total, thirty-two negatives and four contact sheets deal with homecoming POWs and are available online via the Ernst Haas estate/Getty Images website at http://www.gettyimages.at/collections/ernst-haas (accessed 25 October 2018). According to Haas, he designed the layout of the POW article in *Heute* himself, and it is highly likely that Morath wrote the accompanying story.^19^19See Inge Morath correspondence to Hans Weigl, cited in Szeless, ‘“Das Auge lernt und wird sensibler”’. Hans Weigl correspondence, Wienbibliothek Handschriftensammlung, ZPH 847, AB 22.

**Figure 1.**
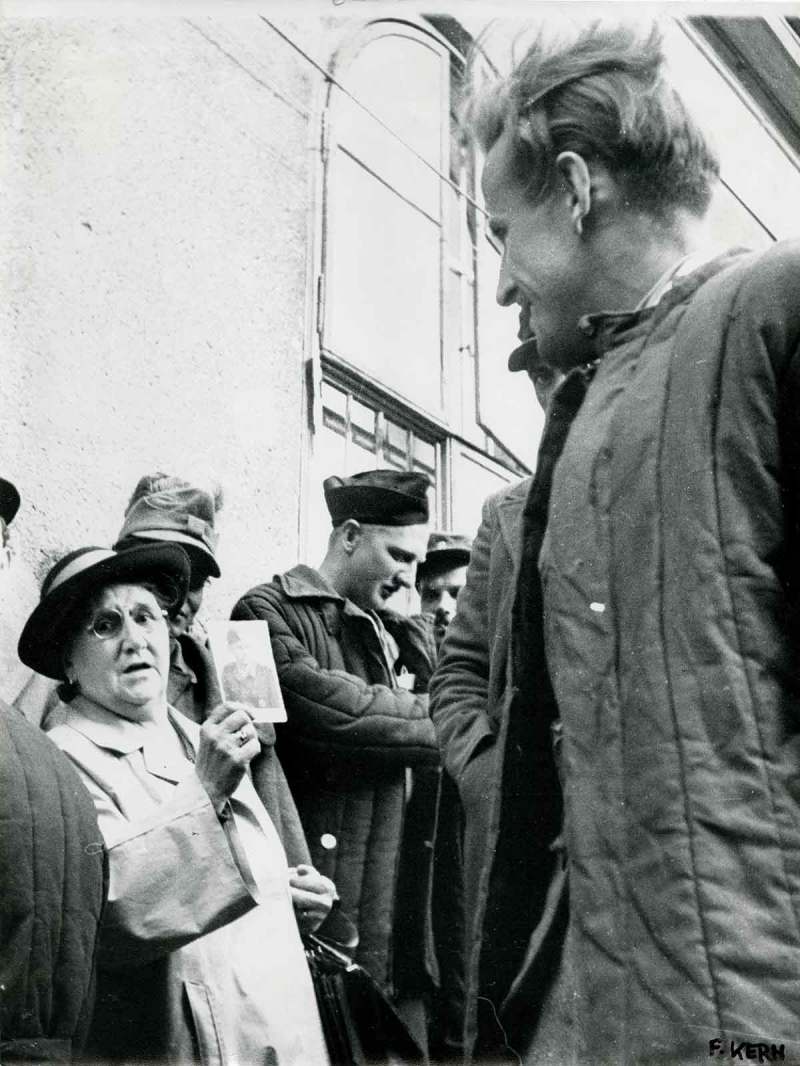
Fritz Kern, *Heimkehrer am Südbahnhof*, 1947, silver gelatin print, 39.6 cm x 29.9 cm. Fotosammlung Ostlicht, Vienna. WL001139.

While constituting a blind spot in the art historical narrative of Haas’s iconic POW photographs, the collaborative aspect of photojournalism, especially the decisive role of the editor or art director, has received much attention in recent scholarship.^20^20Annette Vowinckel, *Agenten der Bilder. Fotografisches Handeln im 20. Jahrhundert*, Göttingen: Wallstein Verlag 2016; Annelie Ramsbröck, Annette Vowinckel, and Male Zierenberg, *Fotografien im 20. Jahrhundert. Vermittlung und Verbreitung*, Göttingen: Wallstein 2013; and *Getting the Picture: The Visual Culture of the News*, ed. Jason Hill and Vanessa R. Schwartz, London: Bloomsbury 2015. As such, Haas’s collaboration with Morath and Trabant and their editorial framing of his pictures deserve closer analysis. Morath provides only a minimum of historical context information in her accompanying text to Haas’s series. She mentions the Südbahnhof in Vienna as the location where these photographs were taken, but neither the Second World War nor National Socialism are referred to except on a visual level, with the lady holding up a photograph of a soldier of the German Wehrmacht. Instead, her text focuses on the waiting women and their emotions. Morath’s text is also used for the photographs’ captions, meaning that words and pictures are closely intertwined; they refer back to one another, intensifying the emotional pull for the reader/viewer and encouraging identification with these fundamental human feelings. She thus clearly understood how to amplify the humanist message of Haas’s pictures and it was her editorial decision and directive to do so. But even with that careful humanist smokescreen, there is still a political edge to Morath’s text since it criticises Stalin’s promise, given in 1947, that all Austrian POWs would be repatriated – a promise which was not kept.^21^21From 1949, transports of returning Austrian POWs from the Soviet Union became increasingly rare despite several petitions from the Austrian government. Stalin’s broken promise triggered huge protests against the Soviet occupying forces on the part of the Austrian population and it is obvious that in 1949, in a journal edited by the US forces and distributed in Austria, this kind of anti-Communist line of attack would be favoured. We therefore highlight a political reading of POW photographs as a topic central to Cold War propaganda.

Trabant was primarily responsible for the international circulation of Haas’s POW photographs. They were published in *Life* magazine within a week of their first appearance and again a month later in the visually progressive Swiss magazine *Du*.^22^22Haas, ‘Last War Prisoners’; and ‘Im Wiener Südbahnhof’, *Du. Kulturelle Monatszeitschrift*, 7 (1949), 24–25. It is highly probable that Haas’s report for *Life* magazine was planned before or at the same time as his report for *Heute*. The preliminary production time in planning issues of *Life* magazine encompassed a few weeks, whereas *Heute* had a shorter and more flexible preliminary production time. We thank Nadya Bair for drawing our attention to this fact. A comparison of Haas’s illustrated articles in *Heute* and *Life* demonstrates the different framing of the topic for European and transatlantic audiences. The US magazine *Heute*, which was distributed in occupied and divided Germany and Austria, addressed an audience familiar and even emotionally and politically involved with the returnees from the Soviet prison camps. An open condemnation of Soviet policy was not feasible in *Heute*, and as such a humanist approach was chosen with Haas’s emotionally stirring photographs printed in large format. The American editors of *Heute* could rely on their upsetting effect and anti-Communist overtones. In *Life*, Haas’s photographs serve the same purpose. With the photographs reduced in size to a double-page spread, the report loses some of its emotional pull and there is a need for more contextual information about Austrian POWs in the accompanying text. The story is much more explicit than *Heute* in its anti-Communist message, with the author concluding: ‘Although Austria claims that some 6400 prisoners of war are still to be accounted for, the grim assumption is that they are dead or lost in the void behind the Iron Curtain’.^23^23Haas, ‘Last War Prisoners’, 31.

Surprisingly, there is hardly any trace of Haas’s POW pictures in local Austrian newspapers and magazines. Only the daily newspaper *Salzburger Nachrichten* published two pictures from the series on its front page on 9 October 1949, but without crediting the photographer.^24^24The credit line under Haas’s photographs in *Salzburger Nachrichten* does not mention the photographer’s name, but credits the Italian magazine *Tempo*. This indicates that Haas’s POW photographs were widely circulated to illustrated magazines elsewhere in Europe. Nevertheless, Austrian magazines and printed materials produced by the US and Soviet occupying forces featured the topic of returning POWs exhaustively. They used photographs taken by local press photographers for their Cold War propaganda, as will be shown in the following section.

## The Historical and Political Context

Since the academic discourse on Haas’s POW series is conspicuous in its lack of historical background information, some facts and figures are appropriate. As already mentioned, after the Second World War, Austria was occupied by the Soviets, the Americans, the British, and the French until the signing of the Austrian State Treaty in May 1955. While the three Western allies released and repatriated Austrian POWs during the first year of the occupation, the Soviets intended to use this foreign workforce, especially those who were well trained, for rebuilding their partially devastated country.^25^25Richard Lein, *Zurück aus dem Krieg. Die Kriegsgefangenen- und Heimkehrerfürsorge der Republik Österreich nach dem 2. Weltkrieg*, Frankfurt: Peter Lang 2006, 90–91. In the first years of the occupation, the Soviets only allowed teenagers, the elderly, and the sick to be repatriated. By 1949, some 136,000 POWs had been released and repatriated to Austria while others – approximately 1,100 – had been sentenced to long-term imprisonment in Soviet labour camps. These so-called ‘late homecomers’ (*Spätheimkehrer*) were only released in the 1950s – some after Stalin’s death in 1953, but most in 1955 as a result of the Austrian State Treaty.^26^26The last transport of Austrian POWs arrived in Wiener Neustadt on 25 June 1955. After this, individual POWs continued to return to Austria. According to the historian Stefan Karner, 156,681 Austrians were held prisoner in the Soviet Union, of whom 10,891 died and 145,790 were repatriated. See Stefan Karner, ‘Die österreichischen Kriegsgefangenen und ihre Arbeit in den sowjetischen Bergwerken 1941–1956’, *Berichte der Geologischen Bundesanstalt*, 41 (1997), 115. Harald Knoll refers here to over 130,000 registered Austrian POWs. See Harald Knoll, ‘Späte Heimkehr. Als Kriegsverbrecher verurteilte österreichische Kriegsgefangene in der Sowjetunion 1944 bis 1953’, in *Kriegsgefangene des Zweiten Weltkrieges. Gefangennahme – Lagerleben – Rückkehr*, ed. Günter Bischof, Stefan Karner, and Barbara Stelzl-Marx, Munich: Oldenbourg 2005, 167. For full details on the repatriation campaigns and on the welfare and care for Austrian POWs, see Lein, *Zurück aus dem Krieg*, 85–131 and 191–99.

Not surprisingly, the most prolific period for photographs of returning POWs by Austrian press photographers were the years 1947, 1949, and 1953–55, when the majority of Austrian POWs were released from the Soviet Union and repatriated. Many of the most prominent Austrian press photographers – among them Karl Votava, the brothers Johann and Fritz Basch, Fritz Kern, Franz Blaha, Karl Franz Schuster, Otto Croy, and Harry Weber – were regularly present at the train station in Wiener Neustadt or at the Südbahnhof in Vienna where transports usually arrived from the Soviet Union or the transfer site in the city of Marmaros-Sziget (in today’s Romania). In fact, the Austrian press photographer Fritz Kern was standing right beside Haas when the latter shot his most famous POW photograph. Kern’s silver gelatin print dates from the year 1947, but was never published (figure 1). Kern pressed the shutter release either a moment before or after Haas had done or would do so; the results are stunningly different. In comparison to Haas’s photograph, the composition of Kern’s photograph is more volatile; the photograph held up by the woman is hard to decipher and the returnee’s face is turned away from the camera in the act of looking at the photograph being shown.

**Figure 2.**
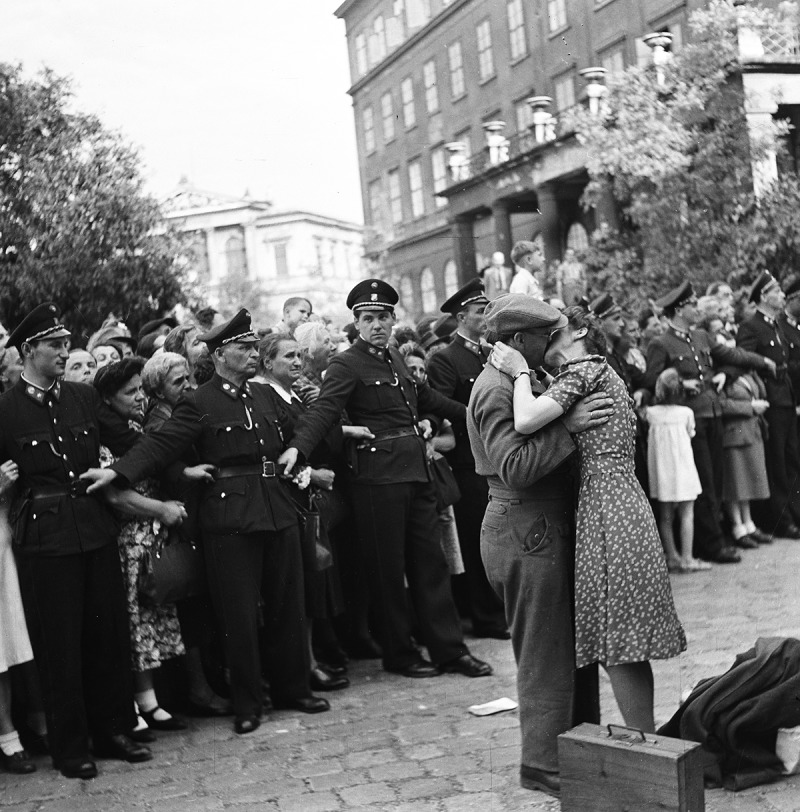
Johann and Fritz Basch, untitled, 1947. Basch Archive held by the newspaper *Oberösterreichische Nachrichten*, Linz.

**Figure 3.**
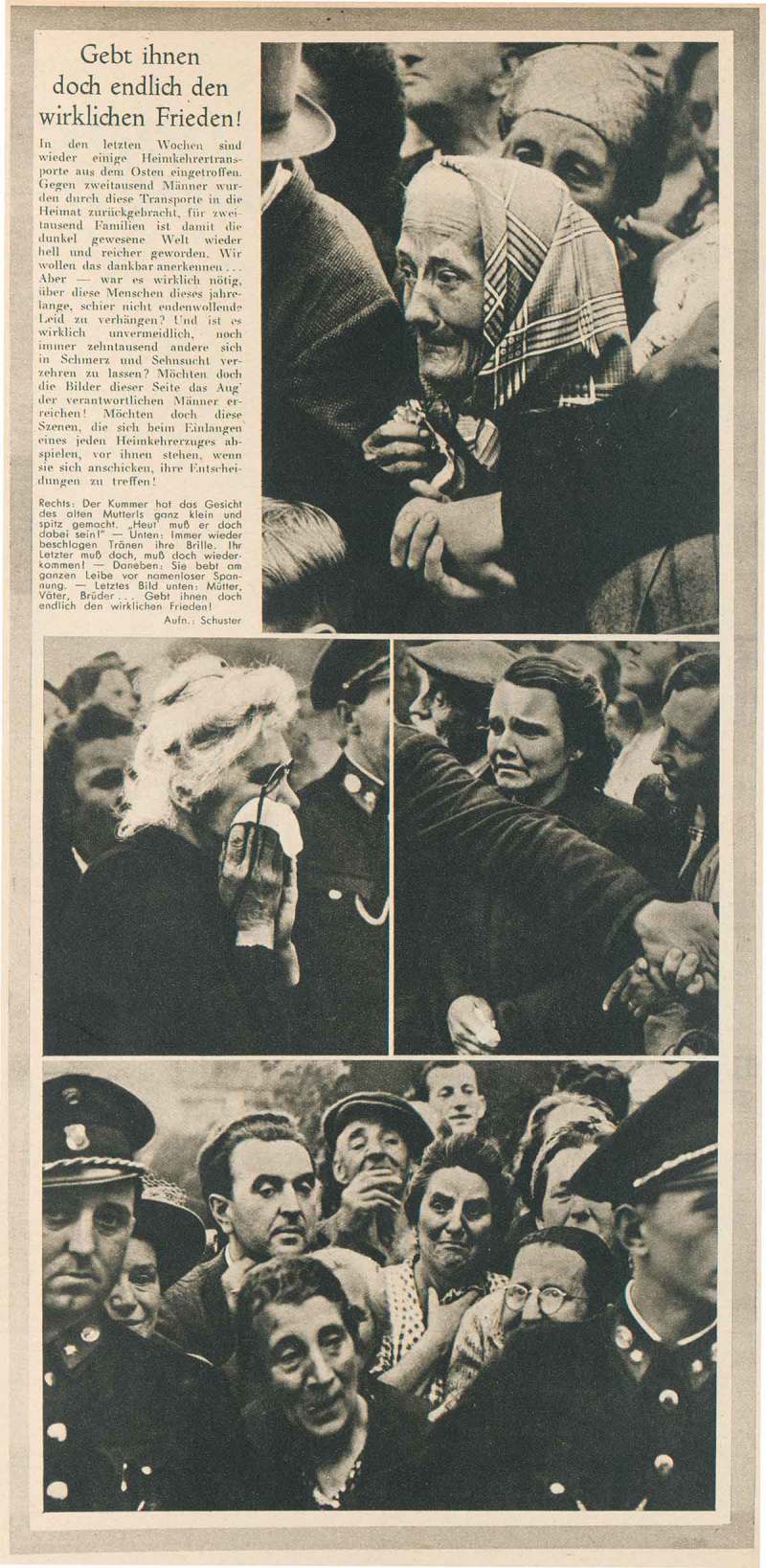
Karl F. Schuster, ‘Gebt ihnen doch endlich den wirklichen Frieden!’, *Bilderwoche* (24 December 1949), n.p. Universitätsbibliothek Wien.

From a total of about four hundred photographs of returning POWs by Austrian press photographers that we were able to locate, a few dominant themes emerge: the waiting crowds, the trains arriving with POWs leaning out of the windows, official speeches by welcoming committees, the hugs, kisses, and tears of reunited partners and families, and onlookers holding signs bearing the names of missing relatives (figure 2).^27^27Research was conducted in Bildarchiv der Österreichischen Nationalbibliothek, Österreichisches Staatsarchiv: Sammlung Berdach, Brandstätter-Imagno, Westlicht Collection, and Salzburg Museum. We also made use of our project database comprising around sixty thousand press photographs taken from the major Austrian illustrated magazines between 1945 and 1955. Many photographs of returning POWs were also published in the magazine *Die Heimkehr. Unabhängige Heimkehrerstimme*, Salzburg, 1947–48. Accordingly, the visual narratives of these events in the illustrated press are very similar. Daily newspapers usually show one or two pictures that epitomise the homecoming, such as an arriving train with waving POWs or a reunited family. There is broad visual coverage of this topic in the four major Austrian illustrated magazines of the period.^28^28The four major postwar journals were *Wiener Illustrierte, Wiener Bilderwoche, Welt-Illustrierte*, and *Große Österreich Illustrierte*. For detailed information on the postwar Austrian illustrated press see Krammer, ‘Rasender Stillstand oder Stunde Null?’, 25–57. ‘We Have Been Waiting For You For So Long’, ‘Home at Last’, and ‘Tears of Joy, Emotion and Despair’ are some of the headlines which, in the lurid manner of a tabloid, repeat the same story again and again.^29^29‘Wir haben schon so auf Euch gewartet’, *Wiener Illustrierte* (20 September 1947), 3 (with photographs by Franz Fink, Franz Votava, and Franz Blaha); ‘Endlich zuhause!’, *Wiener Bilderwoche* (18 September 1947), 2 (with photographs by Franz Blaha, Franz Votava, and Leo Ernst); and ‘Tränen der Freude, der Rührung und der Verzweiflung’, *Arbeiter-Zeitung* (16 October 1953), 3. The overall theme and individual motifs of these reports are very similar to Haas’s account in *Heute*. However, the tabloid versions lack the generous layout, the narrative cohesion, and the bold close-up photographs of Haas’s article. Only one other Austrian press photographer, Karl Franz Schuster (1922–55), seems to have been directly influenced by Haas’s work. Schuster’s report, ‘Give them True Peace at Last!’, was published in *Wiener Bilderwoche* only three months after Haas’s account appeared in *Heute* (figure 3).^30^30Karl Franz Schuster, ‘Gebt ihnen doch endlich den wirklichen Frieden’, *Wiener Bilderwoche* (24 December 1949), 3. See also Simak, ‘Der Photograph Ernst Haas’, 14–16. The similarities are striking, with the same use of close-ups and tight cropping of the anxiously waiting women in order to isolate individual facial expressions. Schuster’s illustrated article focuses even more on the awe and suffering of the waiting women without the emotional denouement of reunion.

**Figure 4.**
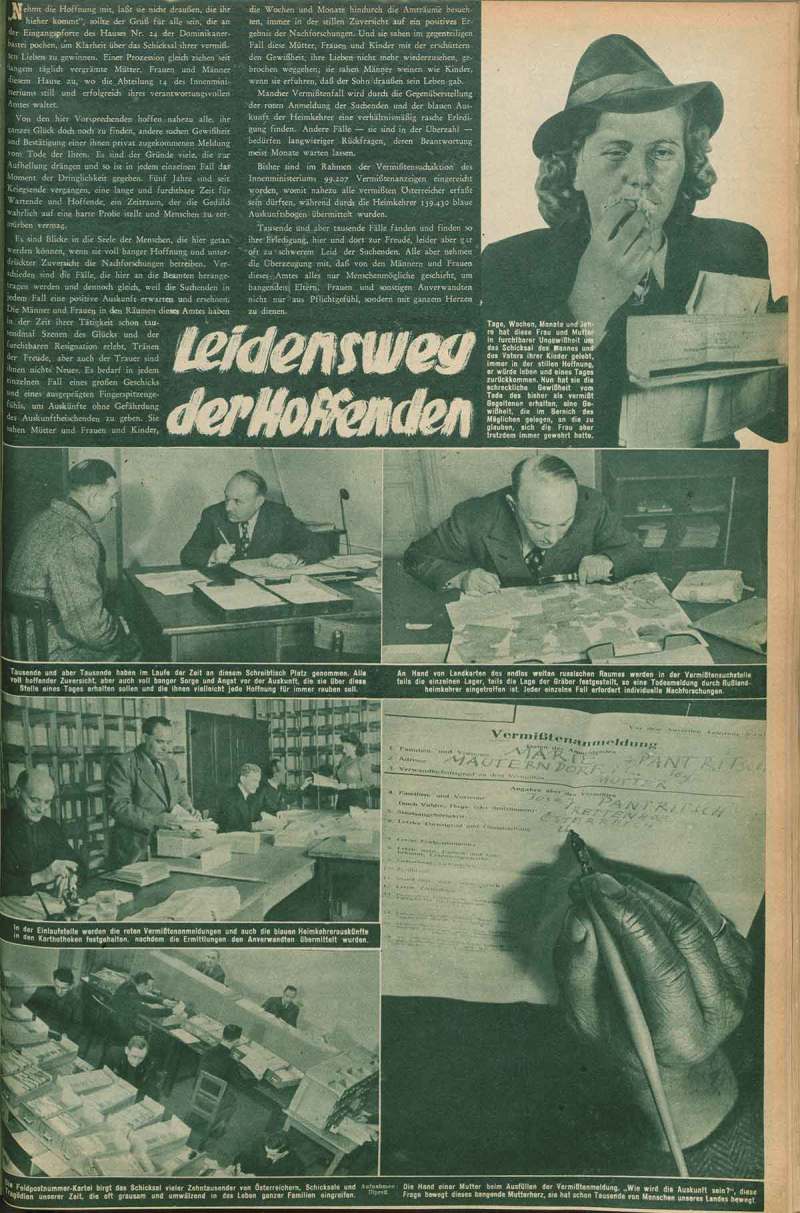
Illpreß, ‘Leidensweg der Hoffenden‘, *Große Österreich Illustrierte* (23 July 1949), 7. Universitätsbibliothek Wien.

What was being done by Austrian politicians to help these women? There were numerous official negotiations with Russia, delegations that travelled to Moscow to lobby for the release of Austrian POWs, and regular transports taking food to Austrian citizens in Russian labour camps.^31^31Lein, *Zurück aus dem Krieg*, 96–101. At a pictorial level, there was little trace of these official efforts in the illustrated magazines, apart from one report in *Große Österreich Illustrierte* of 23 July 1949. The article describes the ‘Leidensweg der Hoffenden’ (Ordeal of the Hopeful), who were in regular contact – often for several years and sometimes without success – with Department 14 of the Austrian Ministry for Interior Affairs.^32^32See *Festschrift anlässlich der Gründung der Stiftung für in Not befindliche ehemalige österreichische Kriegsgefangene*, ed. Rudolf Berdach, Vienna: n.p. 1978. This department was established in order to match missing person reports to the testimonies of returned POWs. The visual protagonist of this article is a missing person’s report form (figure 4). The opening photograph in the top right corner of the page depicts an Austrian woman with a handkerchief pressed to her mouth in despair while, in her other hand, she holds a missing person’s report form. In the lower right corner, an anonymous hand fills out a form, allowing the viewer to decipher the name and address of the missing person. The other four photographs of the narrative take the viewer inside the offices of Department 14 and the consultations between citizens and clerks among the piles of paperwork. Published in a magazine affiliated with the Austrian People’s Party, this report seeks to demonstrate through the combination of text and image that the government is doing its best for the Austrian people. However, this illustrated account is an exception in its attempt to explain the administrative efforts on behalf of a beleaguered and desperate, largely female population.

**Figure 5.**
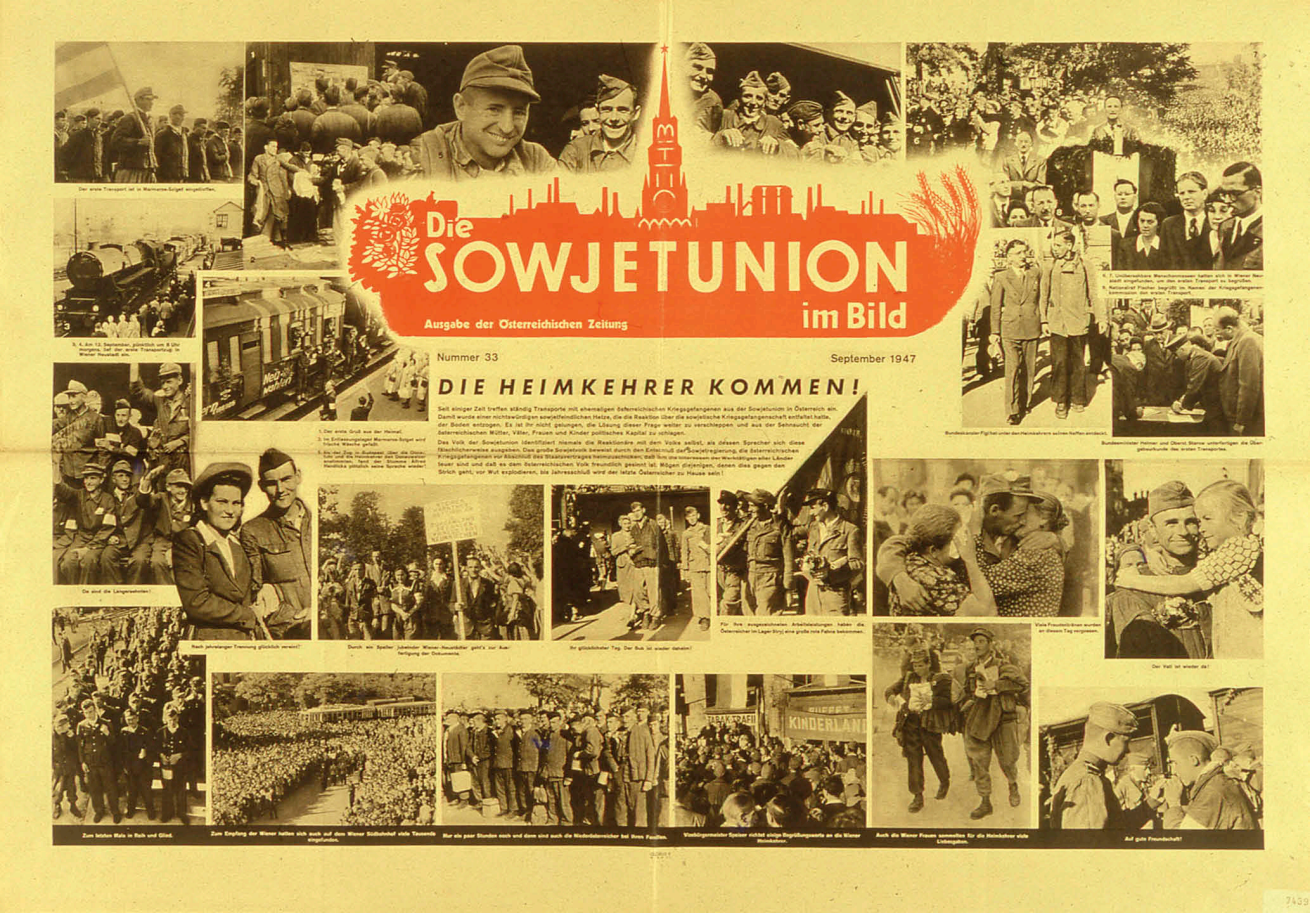
‘Die Heimkehrer kommen!’, *Die Sowjetunion im Bild*, wall newspaper Nr. 33, September 1947. Wienbibliothek im Rathaus, P-7439.

At a visual level, the numerous pictures in the Austrian press of waiting women and their signs bearing the photographs or names of missing family members summarise the tragic story of the uncertain fate of their loved ones. Haas’s famous version of this theme is therefore far from unique, but one among many published accounts illustrated by press photographers of the era.

## The Uses of Imagery

In postwar Austria, the highly emotional topic of returning POWs was prone to political exploitation, not least because the release of Austrian prisoners from the Soviet Union was a diplomatic balancing act beset by setbacks.^33^33The first intervention by the Austrian government on behalf of repatriating Austrian POWs took place on 11 June 1946. The Soviet Union promised to repatriate twenty thousand Austrians, but by Christmas 1946 only 12,500 had been repatriated. Ibid., 25. The Soviet occupation forces in Austria made great efforts to exploit this topic for their own propaganda purposes. In September 1947, the Soviet wall newspaper (poster) *Die Sowjetunion im Bild* announced that ‘The Homecomers are Coming’ and documented these events in over twenty photographs (figure 5).^34^34‘Die Heimkehrer kommen’, *Die Sowjetunion im Bild* (September 1947), wall newspaper. Similarly, there was broad coverage of these homecomers in *Welt-Illustrierte*, the illustrated magazine edited by the Soviet occupation forces.

**Figure 6.**
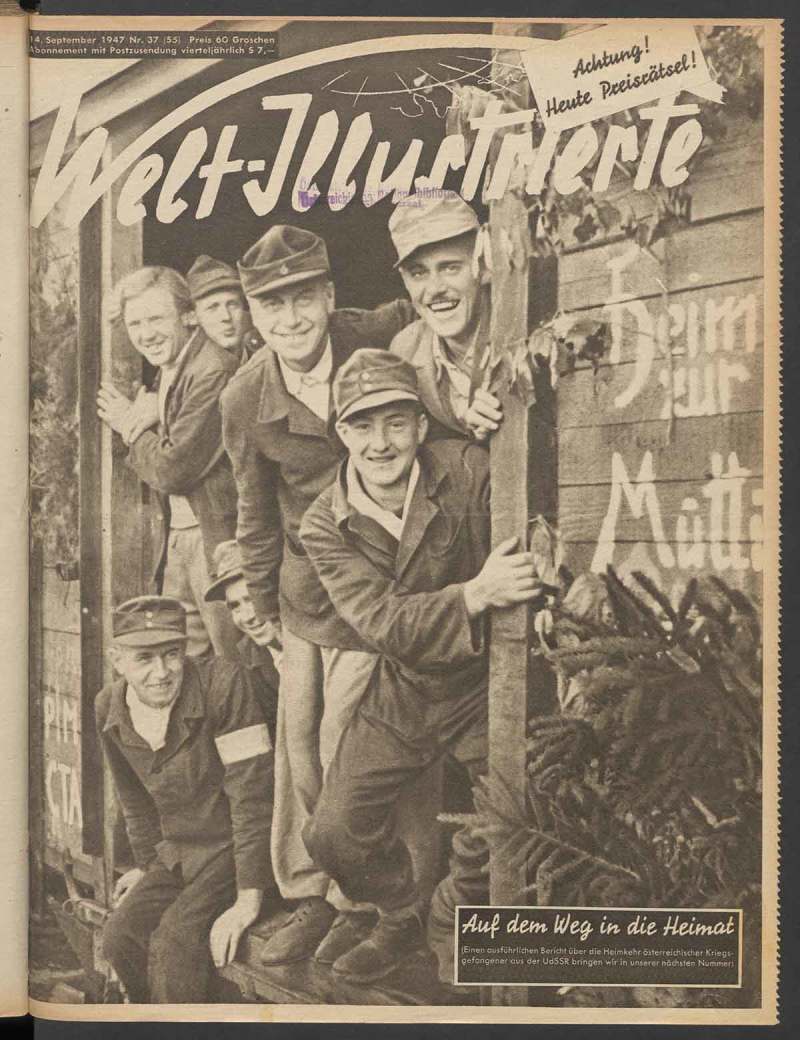
Walter Henisch, ‘Auf dem Weg in die Heimat’, *Welt-Illustrierte* (14 September 1947), front cover. Österreichische Nationalbibliothek, Vienna.

**Figure 7.**
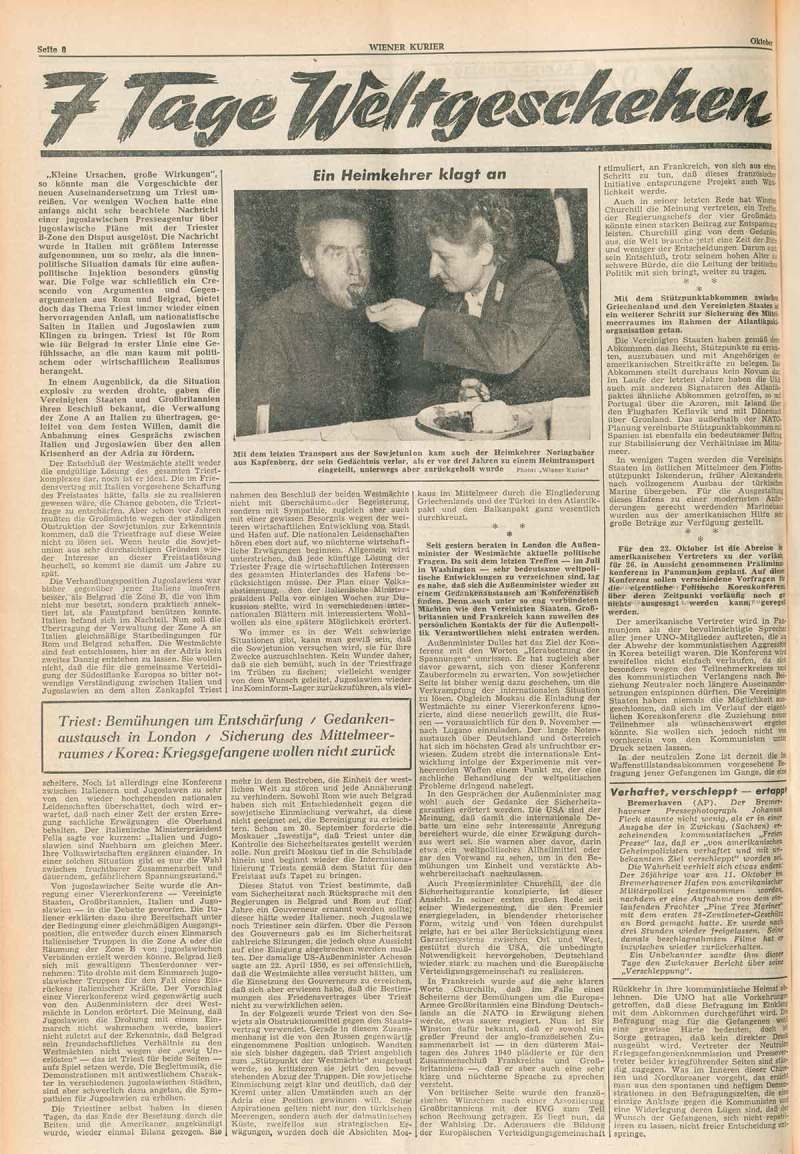
Franz Kraus and Gottfried Rainer, ‘Ein Heimkehrer klagt an’, *Wiener Kurier* (15 October 1953), 8. Universitätsbibliothek Wien.

**Figure 8.**
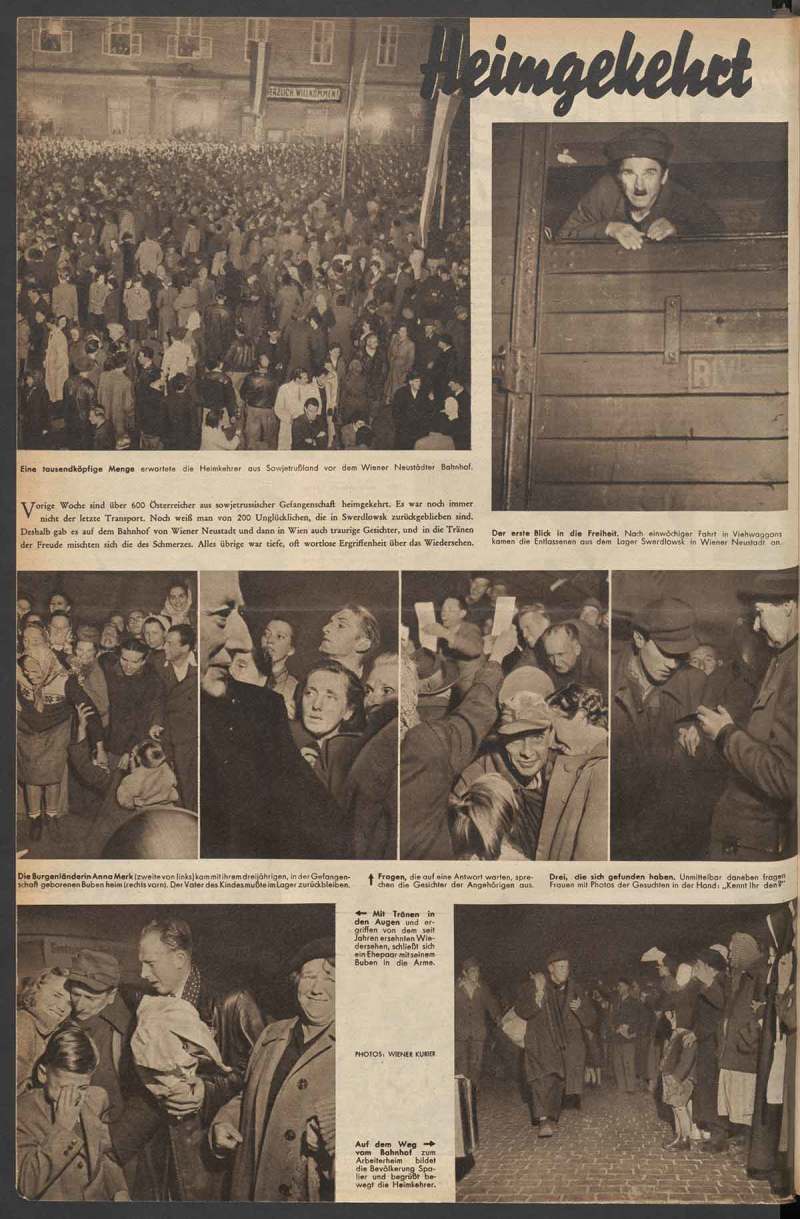
Franz Kraus and Gottfried Rainer, ‘Heimgekehrt’, *Bilderbeilage Wiener Kurier* (24 October 1953), n.p. Österreichische Nationalbibliothek, Vienna.

**Figure 9.**
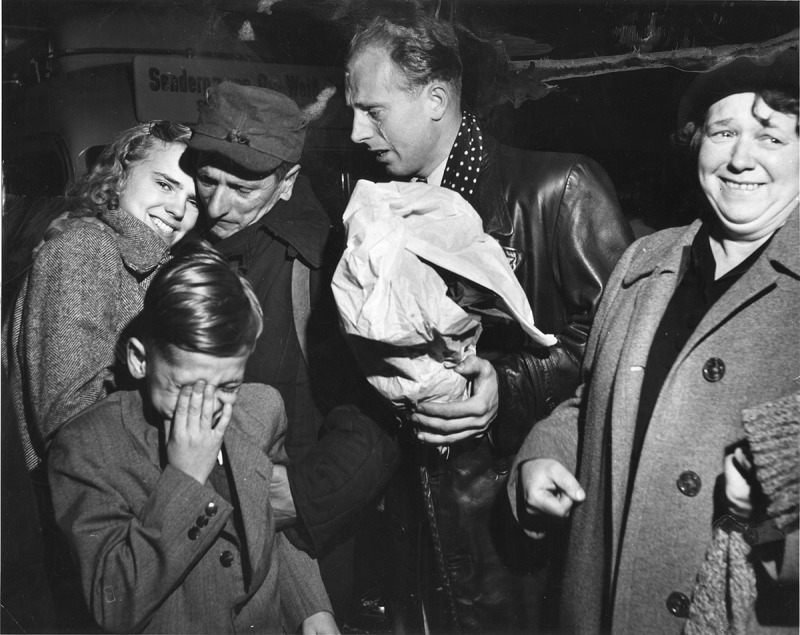
Franz Kraus and Gottfried Rainer, untitled (*Homecomer*), October 1953. National Archives (photo no. 306-PS-55-0974), Washington, DC.

**Figure 10.**
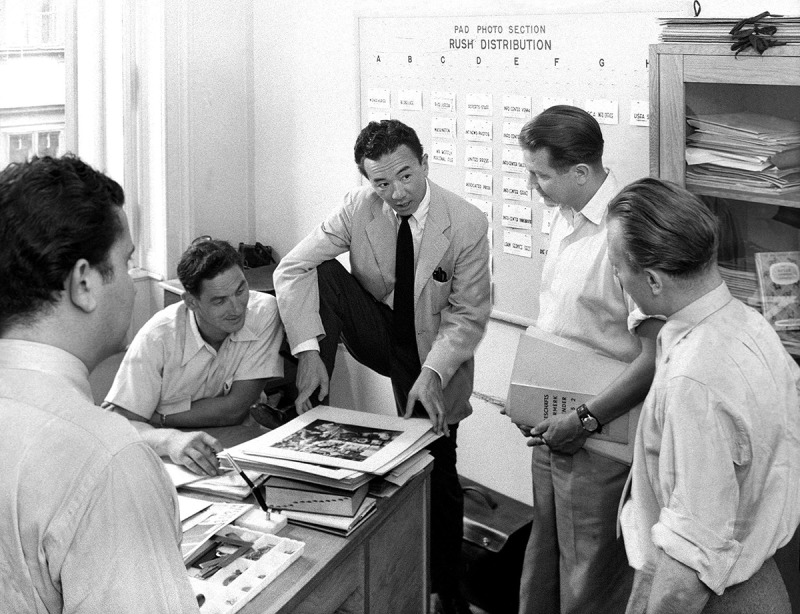
United States Information Services (USIS), untitled (Yoichi Okamoto with staff photographers in the office of *Wiener Kurier*), July 1952. Österreichische Nationalbibliothek, Bildarchiv, US 10.087/9, Vienna.

**Figure 11.**
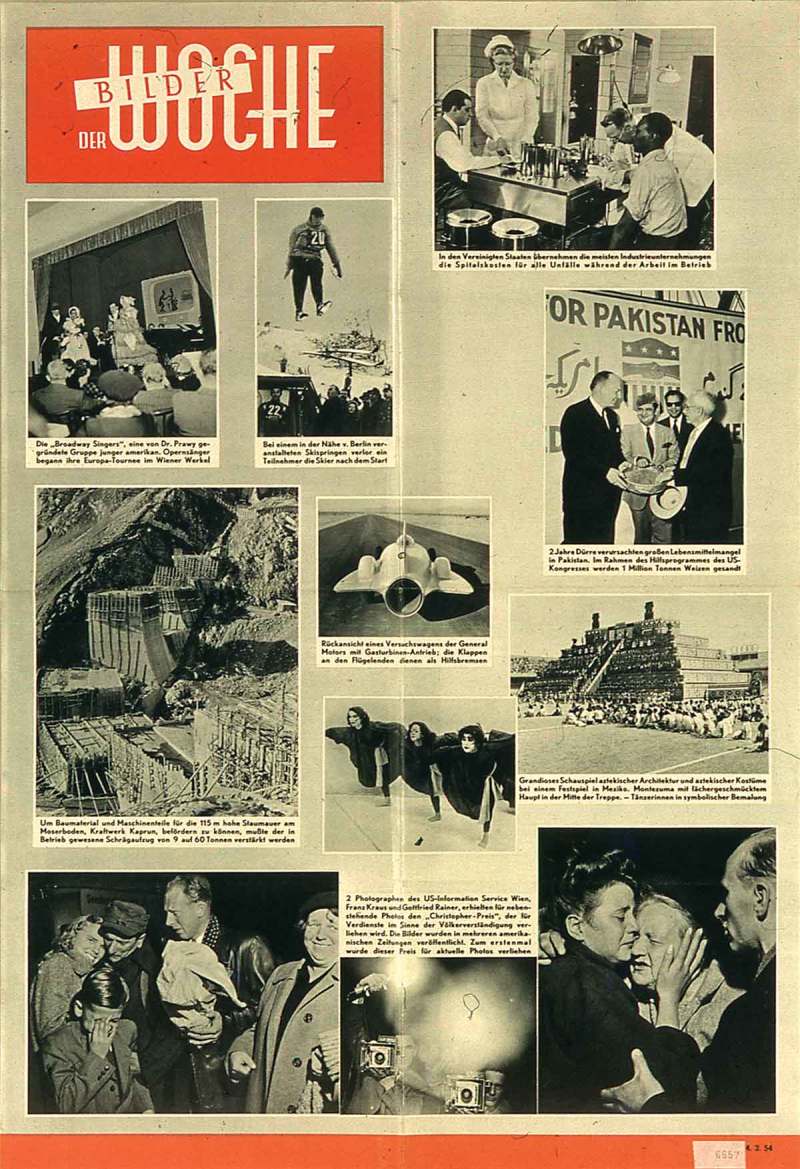
*Bilder der Woche* (4 February 1954), wall newspaper, Wienbibliothek im Rathaus, P6657.

Homecomers were depicted on three covers of *Welt-Illustrierte* in 1947.^35^35Walter Henisch (photographer), ‘Heimgekehrt’, *Welt-Illustrierte* (5 January 1947), cover and 9; ‘Auf dem Weg in die Heimat’, *Welt-Illustrierte* (14 September 1947), cover; and Franz Fremuth and Nova Press (photographer), ‘Wieder daheim’, *Welt-Illustrierte* (21 September 1947), cover and 10–11. If we are to believe these pictures, all of the returning POWs were healthy, young, and happy; they smile from transport wagons on their return journey or reunite with their fiancées (figure 6). The obviously staged photographs of young couples reunited are in stark contrast to the snapshots of crying and embracing family members at the train stations. Readers of *Welt-Illustrierte* learnt about the cultural activities of the POWs in Soviet work camps and about their gratitude to Stalin for having been allowed to contribute to the reconstruction of the Soviet Union. Were these propagandistic distortions really acceptable to Austrian readers at the time? A statement by the Austrian journalist August Beranek provides a potential insight into this question. When assigned to review *Welt-Illustrierte*, Beranek concluded that Communist readers considered it insufficiently progressive whereas indifferent middle-class Austrians viewed it as tendentious political propaganda.^36^36August Beranek, ‘Review and Suggestions for the “Welt-Illustrierte”’ (15 January 1949), Archive of the Ministry of Foreign Affairs of the Russian Federation, Moscow, AVP RF 451/12/253/3, 37–39. After 1948 the retention of Austrian POWs was a source of constant conflict between Austria and the Soviet Union and, accordingly, the topic of returning prisoners was deliberately ignored by *Welt-Illustrierte*.

**Figure 12.**
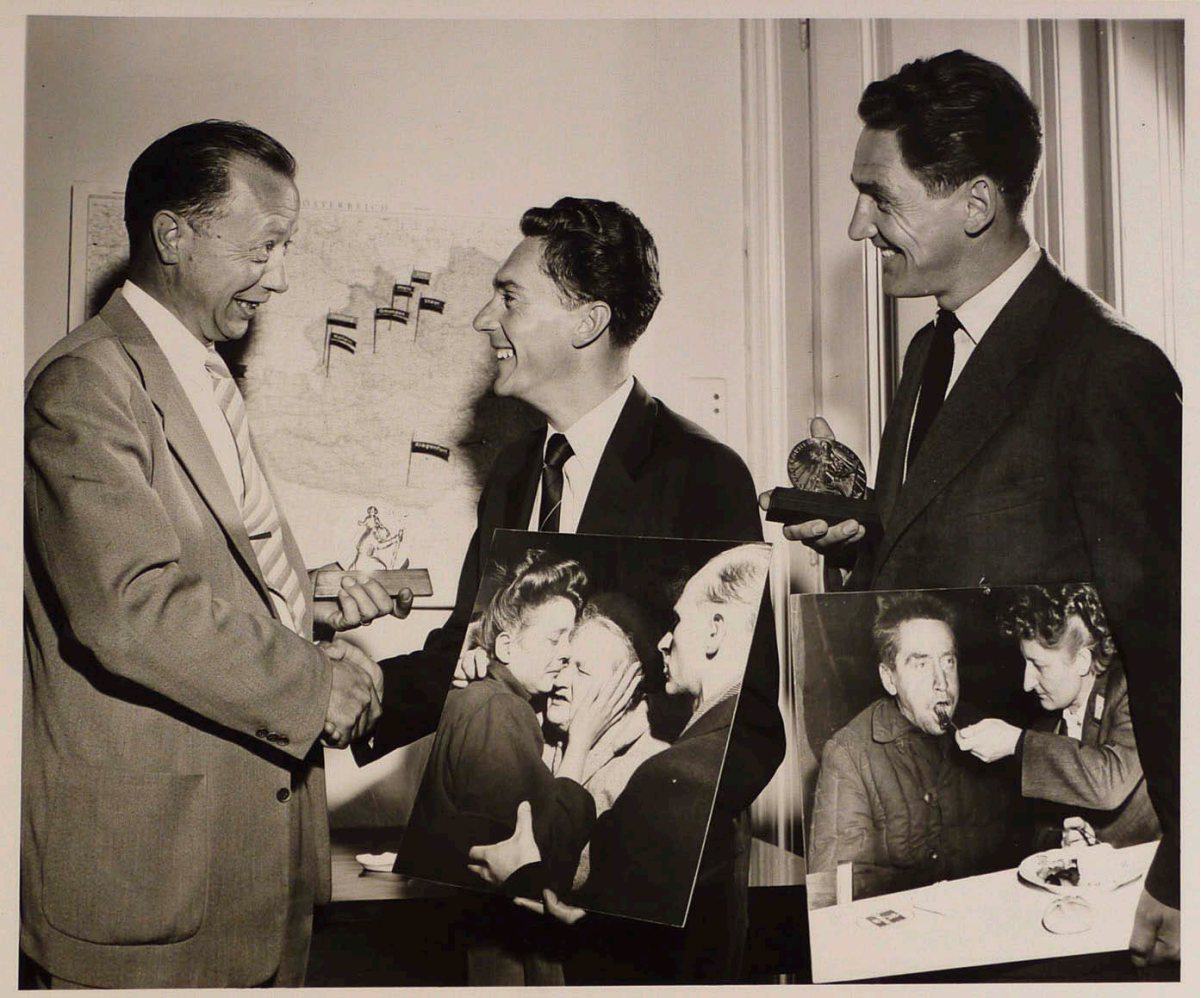
Photographer unknown, untitled (Gottfried Rainer [centre] and Franz Kraus [right] receive the ‘Christopher Award’), 6 June 1954. Österreichische Nationalbibliothek, Bildarchiv, US 12.056, Vienna.

Other Austrian illustrated magazines such as *Wiener Illustrierte, Wiener Bilderwoche*, and *Große Österreich Illustrierte* focused mainly on the emotional aspect of the anxious wait for, or the happy reunion between, long separated family members. Occasionally, these magazines published follow-up stories about a homecomer’s first days at home or a ‘Homecomers’ Hotel’ offering recreation and rehabilitation.^37^37Fritz Kern (photographer), ‘Zurück ins Leben. Die ersten Tage eines Wiener Heimkehrers’, *Wiener Bilderwoche* (25 April 1947), 3; and Fritz Kern (photographer), ‘Heimkehrerhotel im Mölltal’, *Wiener Bilderwoche* (12 February 1948), 7. Otto Croy also undertook a photographic investigation into the situation faced by the homecomers after their arrival in 1946. However, no trace of these pictures in the contemporary illustrated press was found. See Hans Petschar, *Die junge Republik. Alltagsbilder aus Österreich*, Vienna: Ueberreuter 2005, 34–35. In 1953 and 1955, when the so-called late homecomers were released, the narrative changed in the illustrated press. Adopting a more accusatory tone, the headlines emphasised ‘Victims of the Cold War’ and ‘Displaced Persons’ who had been kidnapped from Austria and deported to the Soviet Union.^38^38Karl Franz Schuster, ‘Opfer des kalten Krieges’, *Wiener Illustrierte* (2 July 1955), 7; and ‘Der Zug der Verschleppten’, *Große Österreich Illustrierte* (2 July 1955), 5.

There is also yet another explicitly political context in which images of returning POWs were used in the Austrian press of the postwar years: election campaigns. On 9 October 1949, the day of a general election in Austria, *Salzburger Nachrichten* reproduced two of Haas’s POW photographs on its front page to warn its readers against electoral support for a newly established party, the Wahlpartei der Unabhängigen, which had attracted former National Socialists. In this context, Haas’s photographs were explicitly used as a reminder against fascism and its devastating consequences. The previous day, *Wiener Bilderwoche* had also published a political advertisement for the Socialist Party containing a picture of returning POWs.^39^39‘Wofür am 9. Oktober’, *Wiener Bilderwoche* (8 October 1949), 5–6. In this context, the photograph stressed the merits of the Socialist Party, which, the reader is informed, had been the first party to campaign for the repatriation of Austrian POWs. These versatile deployments of these photographs proved their emotional power and topicality within the context of Austrian domestic politics.

Finally, we turn to an analysis of the coverage of returning POWs in *Wiener Kurier* and its pictorial supplement, *Bilderbeilage*, which was controlled by the US occupying forces and edited by Yoichi Okamoto.^40^40*Wiener Kurier. Zeitschrift der amerikanischen Streitkräfte* first appeared on 27 August 1945. Throughout the Austrian occupation, *Wiener Kurier* was the daily with the highest circulation (between one hundred and fifty thousand and three hundred thousand copies) and thus had a far-reaching influence and enduring impact on Austrian readers. See Krammer, ‘Rasender Stillstand oder Stunde Null?’, 52–56; and Marion Krammer and Margarethe Szeless, ‘“Let’s Hit the Reorientation Line Every Time We Can”: Amerikanische Bildpolitik in Österreich am Beispiel der Pictorial Section’, in *Alliierte Bildpolitik in Österreich 1945–1955*, ed. Marion Krammer, Margarethe Szeless, and Fritz Hausjell, special issue, *Medien & Zeit*, 32:1 (2017), 27–31. While *Wiener Kurier* did not report exhaustively on Austrian POWs returning from the Soviet Union in the 1940s, it provided extensive coverage of the late homecomers in October 1953.^41^41POWs arrived at the train station in Wiener Neustadt on 14 and 19 October 1953. By 1953, the Cold War confrontation between the Soviet Union and the USA had become entrenched as a result of the Korean War. The long overdue release of Austrian POWs from Soviet prison camps undoubtedly played into the hands of US propaganda. The coverage of returning POWs in *Wiener Kurier* and, in particular, the use of captions prove the efficacy of this topic for US propaganda. For example, the photograph of a homecomer paralysed with shock and fed by a helper was published twice in *Wiener Kurier*, once with the reproachful headline: ‘A Homecomer Accuses’ (figure 7).^42^42‘Ein Heimkehrer klagt an’, *Wiener Kurier* (17 October 1953), 8. Another story about a homecomer’s ‘First Christmas in Freedom’ stresses the personal significance of this event having spent ‘a long time away from civilization’.^43^43Yoichi Okamoto, ‘Die ersten Weihnachten in der Freiheit’, *Wiener Kurier* (19 December 1953), picture supplement, 2–3. Although the US approach appears less overt than that of Soviet propaganda, the anti-Communist allusions certainly reached their intended audience.

A report in *Wiener Kurier* about returning POWs warrants close attention. The newspaper sent two staff members from the Pictorial Section, the Austrian press photographers Gottfried Rainer and Franz Kraus, to the train station in Wiener Neustadt – an assignment that provided materials for several richly illustrated stories in *Bilderbeilage* (figure 8).^44^44Kraus and Rainer’s POW photographs were published extensively in *Wiener Kurier* in October 1953: ‘Heimkehrer um 3 Uhr in Wien eingetroffen. Wiedersehen mit Freude und Schmerz’, *Wiener Kurier* (15 October 1953), cover and 3; ‘Ein Heimkehrer klagt an’, *Wiener Kurier* (17 October 1953), 8; and ‘Heimgekehrt’, *Wiener Kurier* (24 October 1953), pictorial supplement, 4. Twelve vintage prints of Kraus and Rainer’s POW series are held by the National Archives and Records Administration, Washington, DC, Still Picture Department, RG 306, PS, Box 533, series 55-9078. ‘Returned Home’ is the simple headline for a pictorially sophisticated story published in the *Wiener Kurier* picture supplement on 24 October 1953. The article consists of eight photographs taken at night at the train station in Wiener Neustadt on the arrival of six hundred POWs from the Soviet Union. The story emphasises the crowds greeting the homecomers and the emotional scenes of reunited family members, accentuated by tight cropping of these photographs. Yet another formal aspect increases the dramatic quality of the pictures; as they were taken at night, the photographers used flash lighting to document the event, capturing faces in various states of expression against the dark background. Furthermore, in three photographs, the subjects look directly at the reader, most evidently in the top right photograph in which a returnee in a livestock wagon takes ‘his first look into freedom’. This direct addressing of the viewer is repeated by another returnee in the middle row, third photograph from the right, as well as by a young woman who embraces her returned husband, reproduced in the bottom left corner. This latter photograph is particularly evocative since it shows the reunion of three generations of one family (figure 9). The crying boy in the foreground, who was probably meeting his father for the first time, reminds viewers of the many years that had separated the family.

This report bears the hallmark of the editor Yoichi Okamoto, who most likely selected the photographs and developed the narrative, but it also epitomises the high standard of press photography achieved by the staff photographers of the American Pictorial Section. When Okamoto took over the Pictorial Section in 1948, he stated: ‘Austrian photographers do not approach picture stories in the “Life” manner. […] Men to fill these qualifications will have to be trained and the training period would take at least six months per candidate’.^45^45Yoichi Okamoto, Memo to Douglas C. Fox, 21 October 1948, National Archives and Record Administration, RG 260, Operations Section, General Records, Box 9, Folder 129. The two Austrian press photographers, Rainer and Kraus, had started working for the Pictorial Section as early as 1948, with Kraus also serving as Okamoto’s personal assistant.^46^46‘Datenbank österreichischer PressefotografInnen 1945–1955’, available at https://datenbankpressefotografie.univie.ac.at (accessed 25 October 2018). By 1953, they had both developed a routine that involved regular editorial meetings with Okamoto, who gave them detailed background information and provided a ‘shooting script’ for each assignment (figure 10). These photographers were technically outstanding and well equipped, as Okamoto proudly asserted: ‘we maintain the best in modern American photographic equipment and furnish the *Wiener Kurier* with pictures that freelance photographers do not attempt, i.e. Strobe-lites and Speed Graphics’.^47^47Yoichi Okamoto, Staff Picture Cost to Wiener Kurier, Memo to Chief of Branch, Ray E. Lee, 15 December 1949. National Archives and Record Administration, RG 260, Pictorial Section, Box 2, Folder 25, Austrian Employees. The exceptional visual quality of the work of the Pictorial Section photographers was the result of intensive training and Okamoto’s professional leadership as photographer, art director, and propaganda officer. Their photographic work was intended to serve the cultural and political mission of the USA in occupied Austria and in Cold War politics. US editors and propaganda officers – Okamoto being foremost among them – made sure that these photographs were published, circulated, and framed in a pro-American manner.

Kraus and Rainer’s photographs fulfilled the criterion for further distribution. In February 1954, two photographs from their series were selected for the US-managed wall newspaper (poster) *Bilder der Woche*, one of which, reproduced in the lower left corner, had previously appeared in the *Wiener Kurier* report discussed earlier (figure 11). The other POW photograph depicts two women, clearly deeply moved, embracing one another. These photographs were chosen for their expressive qualities and symbolic value. Unusually, another image in this report features the two photographers at the train station at night. With their speed-graphic cameras and flash lights, they are about to document the arrival of the POW transport. The caption states in translation: ‘Photographers from the US-Information Service, Vienna, Franz Kraus and Gottfried Rainer, were awarded the “Christopher Award” for promoting mutual understanding between peoples for the photographs shown here. Their pictures were published in several American newspapers. This prize for contemporary photography was awarded for the first time’.

US propaganda officers also decided which pictorial material should be distributed overseas. Okamoto most probably passed on Kraus and Rainer’s POW series to several US newspapers for publication. Similar distribution networks must have been responsible for the photographers’ nomination of this series for the Christopher Award for International Understanding.^48^48The Christopher Award was established in 1949. It is awarded to those active in the field of visual communication by the Christophers, a Christian organisation founded in 1945 by the Maryknoll priest James Keller. In a photograph from the award ceremony, Kraus and Rainer each hold a large print from their series on receiving the Christopher Award in June 1954 (figure 12). The two photographs are of the embracing women featured in *Bilder der Woche*, and the returned POW, paralysed by shock and being fed by a nurse, published in *Wiener Kurier*. The circulation and republication of these photographs in different contexts was a means to bestow them with iconic status and dispense with their initial purpose as propaganda.

The distribution of Kraus and Rainer’s POW series demonstrates that the Cold War was fought at the level of representation. It is certainly not a coincidence that a photograph series dealing with POWs returning from Soviet prison camps was awarded a prize in the USA at the height of the Cold War in 1954. The topic of the late homecomers was given renewed publicity and the implied accusation against the Soviets gained a wider audience. According to Okamoto, this kind of implicit anti-Communist attack was a central strategy of US pictorial propaganda of the era. Okamoto explained quite openly that ‘we do not ridicule the Russians directly; however we will push something like the Berlin airlift to the maximum’.^49^49Yoichi Okamoto, Report on the Activities of the Pictorial Section, n.d. (probably November 1949). National Archives and Record Administration, Washington, DC, RG 260, Pictorial Section, Box 2, Folder 20. Clearly, Austrian POWs returning from Soviet detention were equally suited for such propaganda purposes.

In short, the topic of returning Austrian POWs was highly charged, both emotionally and politically, and was exploited for propaganda purposes not only by the Austrians but by the Soviets and Americans as well. For the USA in particular, the repeated theme of homecomers and the international distribution of Austrian images served to foment resentment against the Soviet Union. The returning POW series by Haas, Rainer, and Kraus are exceptional examples of the use of press photography for this purpose. Their photographs were selected by US propaganda officers and picture editors, distributed internationally and published with an anti-Communist message.

The pivotal contribution made by picture editors in the field of publishing has long lacked sufficient acknowledgement in the history of photography; it is only very recently that a turn towards this topic has been apparent. Nadya Bair, for example, focuses on the picture editors at the global photographic agency Magnum in her project ‘the decisive network’, in which she describes the thematic, political, and commercial considerations that led to the selection and distribution of Magnum photographs in the immediate postwar era.^50^50Nadya Bair, *The Decisive Network: Magnum Photos and the Postwar Image Market*, Berkeley: University of California Press, forthcoming. Drawing on such work, this article has sought to emphasise the role of the picture editors Yoichi Okamoto and Warren Trabant as important co-creators of visual culture and as ‘decisive networkers’ in the field of international postwar photojournalism.

